# Effects of Genetic, Pre- and Post-Harvest Factors on Phenolic Content and Antioxidant Capacity of White Asparagus Spears

**DOI:** 10.3390/ijms10125370

**Published:** 2009-12-16

**Authors:** Eleftherios Papoulias, Anastasios S. Siomos, Athanasios Koukounaras, Dimitrios Gerasopoulos, Evangelos Kazakis

**Affiliations:** 1 Department of Horticulture, Aristotle University of Thessaloniki, 54124 Thessaloniki, Greece; E-Mails: elpapoulias@tellas.gr (E.P.); thankou@agro.auth.gr (A.K.); 2 Department of Food Science and Technology, Aristotle University of Thessaloniki, 54124 Thessaloniki, Greece; E-Mail: dgerasop@agro.auth.gr (D.G.); 3 Department of Agricultural Development, Democritus University of Thrace, 6820 New Orestiada, Greece; E-Mail: bkazakis@agro.duth.gr (E.K.)

**Keywords:** asparagus, cultivars, harvesting season, spear thickness, portion, color, storage, total phenolics, antioxidant capacity

## Abstract

The effects of genetic, pre-harvest (season of harvest, spear diameter, spear portion and spear tip color) and post-harvest factors (storage and domestic preparation practices, e.g., peeling and cooking) on total phenolic, flavonoid and ascorbic acid content of white asparagus spears and their correlation with antioxidant capacity (DPPH and FRAP) were studied. Results showed that genetic material was important for the total phenolic content but not season of harvest, spear diameter or storage. Violet spear tips and apical spear portions showed the largest amount of total phenolics. Peeling did not affect total phenolics in fresh asparagus, whereas it reduced their content in stored asparagus, while cooking resulted in an increase in both fresh and stored asparagus. However, the soluble extract of total phenolics and flavonoids were minor and the missing significance of phenolics and flavonoids in antioxidant capacity of white asparagus spears depends on these small amounts.

## 1. Introduction

Phenolic compounds are secondary metabolites in plants and as such are present in some plant foods. Their functions in plants are not always known, but some are structural polymers, UV screens, antioxidants, attractants and others are involved in non-specific defense mechanisms [1]. Also, it should be mentioned the importance of phenolics in determining some quality attributes and properties in fresh fruits and vegetables, like the color, texture, taste and flavor. One of the principal roles that have been proposed as part of the actions of phenolics in man is that of an antioxidant [1,2].

With respect to the relative phenolic concentration based on dry weight, green asparagus has been ranked fourth among 23 vegetables [3]. In green asparagus, a high correlation between both the antiradical activity and ferric reducing power and total phenolic content was found, suggesting that phenolics could be mainly responsible for both activities [4]. Among the vegetables commonly consumed in Italy [5] and USA [3] green asparagus had the greatest antioxidant capacity.

Phenolic accumulation in plants can be affected by genetic factors, environmental and cultural conditions and also various stresses [1]. The sample origin and cultivar were important factors for the total phenolic content of both green and white asparagus spears [4].

Contradictory data have been reported concerning the effect of cooking process on phenolic compounds of vegetables [6]; it has been proposed that this effect is strongly dependent upon the vegetable species and the cooking conditions [7]. According to a recent study [8], total phenolic content of green asparagus was increased after boiling.

However, most of the available information on asparagus phenolic content and its antioxidant capacity has been focused on the green asparagus [4,8,9]. It is important to mention that there are many differences on physiology and composition of green and white asparagus [10,11] since in green asparagus, the spears are grown in the presence of sunlight, while white asparagus is produced in the absence of sunlight. The traditional growing method is to mound up soil over the plant row, before the spears start to grow and harvesting is done before the spear is exposed to light [12].

Color of the tips and spear diameter are quality characteristics according to specifications for grades and standards imposed by the Commission Regulation (EEC) No 454/92 [13] and its amendments. Thin spears or spears with a violet color in the tip, irrespectively of their size, are judged to be of a lower quality [12]. Traditionally, white asparagus is commercialized as whole spears, but lately, spears have been developed as a minimally processed product following peeling and segmentation [14].

Therefore, the objectives of the present study were to determine the total phenolic, total flavonoid and ascorbic acid content (which are the major antioxidants) of white asparagus spears in relation to genetic material, season of harvest, spear diameter, spear portion, spear tip color, storage, and domestic preparation practices and to correlate it with antioxidant capacity.

## 2. Results

### 2.1. Effect of Cultivar

Cultivar significantly affected all the measured parameters. The highest values were recorded for total phenolics in ‘Dariana’ and ‘Darlisa’, for total flavonoids in ‘Larac’, for ascorbic acid in ‘Darsiane’, for DPPH in ‘Darlisa’ and ‘Dariana’ and for FRAP in ‘Larac’, ‘Dariana’ and ‘Darlisa’ (Table 1). For total phenolics, total flavonoids and FRAP differences were not significant among the other cultivars, while ‘Stelina’ and ‘Darbella’ exhibited the lowest ascorbic acid content and ‘Darbella’, ‘Steline’ and ‘Cipres’ the lowest DPPH.

### 2.2. Effect of Harvesting Season

Season of harvest significantly affected all the measured parameters with the exception of total phenolic content, that ranged from 0.227 to 0.312 mg gallic acid equivalents/g f.w. (Table 2). A significant fluctuation in total flavonoid content was observed throughout the harvesting season with its peak (0.221 mg rutin equivalents/g f.w.) in mid season. Ascorbic acid content and FRAP were found to be highest at the first half of the harvesting season, while the opposite was observed for DPPH.

### 2.3. Effect of Spear Diameter

Spear diameter significantly affected only ascorbic acid content (Table 3) and a linear decrease of ascorbic acid content with increasing spear diameter was observed (r =−0.867, *P* < 0.0053).

### 2.4. Effect of Tip Color and Spear Portion

In both white and violet spears, apical portions (0–7 cm from the tip) were found to be richest in all bioactive compounds and measured antioxidant capacity (Table 4). Significant differences between middle (7–14 cm from the tip) and basal (14–21 cm from the tip) portions were observed only in violet spears in total phenolic and ascorbic acid content. All the measured parameters were found to be higher in the apical portions of the violet spears than the white ones.

### 2.5. Effect of Storage and Domestic Preparation

Storage significantly affected only total flavonoid and ascorbic acid content, resulting in an increase from 0.179 to 0.292 mg rutin equivalents/g f.w. and in a decrease from 19.33 to 15.86 mg/100 g f.w., respectively (Table 5). It should be mentioned that at the end of storage, the spears lost 3.27 ± 0.36% of their initial fresh weight. Peeling of fresh asparagus resulted only in a significant decrease of ascorbic acid content, while peeling of stored asparagus resulted in a significant decrease of total phenolic and total flavonoid content. The spears subjected to peeling had 78.86 ± 1.42% of the weight of the unpeeled spears. Due to cooking, the spears lost 10.33 ± 0.43% of their initial fresh weight. Furthermore, cooking of both fresh and stored asparagus increased total phenolic content, DPPH and FRAP antioxidant capacity. Spear cooking resulted in an increase of DPPH antioxidant capacity by 196 and 192% in fresh and stored at 2 °C for six days asparagus, respectively, and of FRAP antioxidant capacity by 259 and 218% in fresh and stored asparagus, respectively (Table 5). No significant differences between fresh and stored asparagus after cooking were observed.

### 2.6. Correlation of Total Phenolic Content and Antioxidant Capacity

A significant linear relationship between total phenolic content and antioxidant capacity was observed only in the spear portion and domestic preparation practices experiments for both DPPH and FRAP and in the spear diameter experiment for DPPH. The highest correlation (r = 0.933, *P* < 0.0000*** and r = 0.888, *P* < 0.0000***, for DPPH and FRAP, respectively) was found in the spear portion experiment with violet spears. In these cases, the correlation of total phenolic content was higher with DPPH than with FRAP.

On the other hand, total flavonoids were significantly correlated with antioxidant capacity only in the spear portion and cultivar (cv. ‘Darlisa’) experiments for both DPPH and FRAP and in the harvesting season and cultivar (cv. ‘Darsiane’) experiments for FRAP, while ascorbic acid was significantly correlated with antioxidant capacity in the harvesting season and spear portion experiments for both DPPH and FRAP and in cultivar (cv. Darbella) experiment for FRAP. The highest correlation for both total flavonoids (r = 0.960, *P* < 0.0000*** and r = 0.945, *P* < 0.0000***, for DPPH and FRAP, respectively) and ascorbic acid (r = 0.969, *P* < 0.0000*** and r = 0.947, *P* < 0.0000***, for DPPH and FRAP, respectively) was found in the spear portion experiment with violet spears. In these cases also, the correlation of total phenolic content was higher with DPPH than with FRAP.

## 3. Discussion

Among the factors studied in the present work genetic material as well as spear portion and spear tip color appeared to be important for the total phenolic content of white asparagus spears, but not season of harvest and spear diameter (Tables 1–4). Genetic material has also been reported to be an important factor affecting the phenolic content of green asparagus [4,8,9]. Phenolic content is considered an important characteristic in selecting breeding lines that show high antioxidant capacity and its determination might make the screening progress relatively easy [9].

Violet spear tips and apical spear portions (0–7 cm) showed the largest amount of total phenolics (Table 4), implying that exposure to light is essential for its accumulation and that these compounds are mainly located at the upper part of spears. These findings also suggest that the distribution of phenolics is not related to that of fiber content of spears, since the thin spears and the more fibrous lower spear portions did not had higher phenolic content than the thicker spears and the upper spear portions. Similar results were observed by comparing violet and white spears with a length of 24 and 17 cm, respectively [9] as well as upper (0–11 cm) and lower (11–22 cm) spear portions of both green and white spears [11].

On the other hand, total phenolic content was highly influenced by domestic preparation practices (peeling and cooking) but not by storage. Peeling did not affect total phenolic content of fresh asparagus, whereas it reduced by 14.1% total phenolic content of asparagus stored at 2 °C for six days. Spear cooking resulted in an increase of total phenolic content by 30.6 and 31.2% in fresh and stored asparagus, respectively (Table 5). These results are very consistent with the findings of Fanasca *et al*. [8] who found a 32% increase in total phenolics after cooking on green asparagus when compared to fresh one. However, both positive and negative effects have been reported in other vegetables depending upon differences in processing conditions and morphological and nutritional characteristics of vegetable species. In broccoli and carrots, cooking reduced the total phenolic compounds [7].

Genetic material as well as harvesting season, spear portion and spear tip color appeared to be important pre-harvest factors for the antioxidant capacity (DPPH and FRAP) of white asparagus spears, but not spear diameter (Tables 1–4). Moreover, antioxidant capacity was highly influenced by cooking process but not by storage. These results are consistent with the findings of Fanasca *et al*. [8] who found a 20.1% increase in antioxidant capacity after cooking of green asparagus. In other vegetables such as broccoli and carrots, cooking also increased the antioxidant capacity, probably because of matrix softening and increased extractability of compounds, which could be partially converted into more antioxidant chemical species [7]. During processing, loss of antioxidants or formation of compounds with pro-oxidant action may lower the antioxidant capacity. On the other hand, alterations to the structure of the existing antioxidants, as well as the formation of novel antioxidant components may enhance the initial antioxidant status [18]. Thus the influence of processing may be positive, negative, or none.

There are very few recent studies on the impact of common domestic practices on flavonoid content of plant foods and it appears that there are some noteworthy discrepancies in the conclusions that have been drawn. It was found that treatments such as chopping might cause moderate changes to flavonol composition, but the overall impact is rather without particular importance [19]. However, boiling for 60 min resulted in 43.9% decrease in total flavonol content for green asparagus and it was also observed that boiling extracted a considerable amount of conjugates into the cooking water [19]. In our study, only peeling of stored white asparagus had a significant effect, decreasing total flavonoid content (Table 5).

Ascorbic acid content of the white asparagus spears was not affected by cooking process (Table 5) and this is consistent with the findings of Miglio *et al*. [7] who also reported no significant losses in courgettes (*Cucurbita pepo* L.) after boiling, although it is well known that cooking is often responsible for the greatest loss of ascorbic acid in vegetables and that the extent of the loss depends on cooking method and its duration [20]. Boiling green asparagus spears significantly reduced the total ascorbate by 52% [8] and an explanation for this disagreement could be the shorter time of boiling used in our study (10 in comparison to 15 min).

The soluble extract of total phenolics and flavonoids, as antioxidants, measured in the present study in white asparagus, were minor, when compared to green asparagus [4,8,9,11], whereas ascorbic acid could have interest, being its average amount around a half of that of green asparagus [11]. Only apical portion, and especially those from violet spears, had interesting antioxidant activity (Table 4), since antioxidants are one of the plant responses to an external stress, especially to UV radiation [1].

Only in a few cases in our experiments the antioxidant capacity of white asparagus spears was significantly correlated with total phenolic or flavonoid content. However, in green asparagus, a high correlation between both the antiradical activity and ferric reducing power and total phenolic content was found, suggesting that phenolics could be mainly responsible for both activities [4]. Moreover, recent evidence strongly suggests that the antioxidant capacity of plant foods and products may be attributed, to a great extent, to their content of compounds of flavonoid nature [16,17]. Missing significance of phenolics and flavonoids in antioxidant capacity of white asparagus spears depends on these small amounts and values. Therefore, spears do not receive sunlight during growth, so white asparagus could be considered as a vegetable with low antioxidant capacity, because of its low content of phenolics and flavonoids.

## 4. Experimental Section

### 4.1. Plant Material

To study the effect of cultivar, white asparagus (*Asparagus officinalis* L.) spears of eight cultivars (‘Darbella’, ‘Dariana’, ‘Darlise’, ‘Darsiane’, ‘Grolim’, ‘Cipres’, ‘Larac’ and ‘Steline’) were morning harvested in the middle of the harvesting period from ridged plants in an 5-year-old experimental plantation at Tichero, Thrace, Greece and immediately placed into lidded styrofoam containers to avoid light exposure. After 6 h transportation in darkness at ambient temperature, the spears were trimmed to 21 cm in length and washed thoroughly. Straight and undamaged spears, with white closed bracts and a mid diameter of 16.0–19.9 mm were selected. The experiment was repeated at the end of harvesting period.

For the other experiments, white asparagus spears of the cultivar ‘Grolim’ were morning harvested from ridged plants in an 8-year-old commercial plantation at Galatades, Macedonia, Greece and immediately placed into lidded styrofoam container to avoid light exposure. After 3 h transportation in darkness at ambient temperature, the spears were trimmed to 21 cm in length and washed thoroughly. Straight and undamaged spears, with closed bracts were selected.

To study the effect of harvesting season, four harvests were done through the 40-day harvesting period at 10, 20, 30 and 40 days after the beginning of harvest and spears with white tips and a mid diameter of 16.0–19.9 mm were used.

To study the effect of spear diameter, spears with white tips harvested at the beginning of the harvesting period were classified in four classes, according to their diameter at the mid of the spear: 12.0–15.9, 16.0–19.9, 20.0–23.9 and 24.0–27.9 mm. The experiment was repeated at the end of harvesting period.

To study the effect of spear tip color and spear portion, spears with white and violet tip and a mid diameter at 16.0–19.9 mm were harvested at the beginning of the harvesting period and were cut at three portions: apical (0–7), middle (7–14) and basal (14–21 cm from the tip). The experiment was repeated at the end of harvesting period.

To study the effect of storage and domestic preparation practices (peeling and cooking) spears with white tips and a mid diameter at 16.0–19.9 mm were harvested at the beginning of the harvesting period and were peeled with a sharp vegetable peeler starting 3 cm below the tip and then boiled for 10 min in 2 L of tap water or were stored at 2 °C for 6 days and then peeled and boiled. The experiment was repeated at the end of harvesting period. In each experiment, three replicates were used for each treatment, with 7–10 spears per replicate.

### 4.2. Determinations

Spears of each replication were macerated in a blender for the determination of total phenolic, total flavonoid and ascorbic acid content as well as of DPPH radical scavenging activity and FRAP. Total phenolic content was determined according to the method of Folin-Ciocalteu reaction [21], using gallic acid as standard. Total flavonoid content was determined according to Zhishen *et al*. [22] and the results were expressed as mg rutin equivalent per gram fresh weight. Ascorbic acid was extracted in 1% oxalic acid and measured by using Reflectoquant ascorbic acid test strips and an RQflex portable reflectometer (Merck, Darmstadt, Germany). DPPH radical scavenging activity was determined using a modified method of Brand-Williams *et al*. [23]. Sample homogenate, 5 g, was extracted with 25 mL methanol in ice, centrifuged at 5,000 g for 10 min and filtered through Whatman No. 1 paper. The supernant was adjusted with methanol to 25 mL. Extract, 50 μL, was added to 2,950 μL of 100 μM methanolic DPPH, vortexed and kept in the dark at room temperature. The decrease in absorbance of the resulting solution was monitored at 517 nm for 30 min. Ascorbic acid was used as a standard and DPPH radical scavenging activity were expressed in mg of ascorbic acid equivalents antioxidant capacity per gram fresh weight. The antioxidant capacity by FRAP assay was determined by the method of Benzie and Strain [24]. The results were expressed as μM FRAP per gram fresh weight.

### 4.3. Statistical Analysis

A completely randomized design was used. Data from the repeated experiments were pooled and analysis of variance (ANOVA) was performed using the MSTAT version 4.00/EM (Michigan State University). Means were separated by Duncan’s multiple range test at the 0.05 level.

## 5. Conclusions

The results of the present study indicate that genetic material was important for the total phenolic content of white asparagus spears but not season of harvest, spear diameter or storage. Violet spear tips and apical spear portions showed the largest amount of total phenolics. Peeling did not affect total phenolics in fresh asparagus, whereas it reduced their content in stored asparagus, while cooking resulted in an increase in both fresh and stored asparagus. However, the soluble extract of total phenolics and flavonoids were minor, when compared to green asparagus and the missing significance of phenolics and flavonoids in antioxidant capacity of white asparagus spears depends on these small amounts.

## Figures and Tables

**Table 1.** Total phenolic (mg gallic acid equivalents/g f.w.), total flavonoid (mg rutin equivalents/g f.w.), ascorbic acid (mg/100 g f.w.) content, DPPH (mg ascorbic acid equivalents antioxidant capacity/100 g f.w.) and FRAP (μM/g f.w.) of eight cultivars of white asparagus spears.

**Cultivar**	**Total phenolics**		**Total flavonoids**		**Ascorbic acid**		**DPPH**		**FRAP**	
Darbella	0.302	b	0.177	b	15.33	cd	5.26	c	15.88	c
Dariana	0.378	a	0.176	b	17.86	b	10.93	a	22.82	ab
Darlisa	0.341	ab	0.171	b	18.16	b	11.99	a	22.79	ab
Darsiane	0.311	b	0.169	b	20.19	a	8.76	b	17.17	c
Grolim	0.328	b	0.176	b	17.82	b	8.12	b	15.45	c
Cipres	0.325	b	0.185	b	17.28	b	6.51	c	18.50	bc
Larac	0.324	b	0.242	a	16.92	bc	9.01	b	25.10	a
Steline	0.328	b	0.181	b	14.10	d	6.28	c	19.29	bc

Each value is the mean of six replicates. Different letters at each column denote significant differences between means (Duncan’s multiple range test, P < 0.05).

**Table 2.** Total phenolic (mg gallic acid equivalents/g f.w.), total flavonoid (mg rutin equivalents/g f.w.), ascorbic acid (mg/100 g f.w.) content, DPPH (mg ascorbic acid equivalents antioxidant capacity/100 g f.w.) and FRAP (μM/g f.w.) of white asparagus spears (cv. ‘Grolim’) during a 40-day harvesting period.

**Harvesting season (days)**	**Total phenolics**	**Total flavonoids**		**Ascorbic acid**		**DPPH**		**FRAP**	
10	0.227	0.181	b	19.87	ab	4.67	b	17.13	a
20	0.262	0.221	a	20.76	a	4.77	b	16.59	a
30	0.312	0.131	c	18.94	b	6.99	a	10.14	b
40	0.274	0.177	b	18.78	b	7.61	a	11.42	b

Each value is the mean of three replicates. Different letters at each column denote significant differences between means (Duncan’s multiple range test, P < 0.05).

**Table 3.** Total phenolic (mg gallic acid equivalents/g f.w.), total flavonoid (mg rutin equivalents/g f.w.), ascorbic acid (mg/100 g f.w.) content, DPPH (mg ascorbic acid equivalents antioxidant capacity/100 g f.w.) and FRAP (μM/g f.w.) of white asparagus spears (cv. ‘Grolim’) as affected by spear diameter.

**Spear diameter (mm)**	**Total phenolics**	**Total flavonoids**	**Ascorbic acid**		**DPPH**	**FRAP**
12.0–15.9	0.259	0.156	21.55	a	6.17	13.70
16.0–19.9	0.251	0.179	19.33	b	6.14	14.28
20.0–23.9	0.236	0.172	16.83	c	5.44	14.98
24.0–27.9	0.251	0.152	16.00	c	6.39	14.56

Each data is the mean of six replicates. Different letters at each column denote significant differences between means (Duncan’s multiple range test, P < 0.05).

**Table 4.** Total phenolic (mg gallic acid equivalents/g f.w.), total flavonoid (mg rutin equivalents/g f.w.), ascorbic acid (mg/100 g f.w.) content, DPPH (mg ascorbic acid equivalents antioxidant capacity/100 g f.w.) and FRAP (μM/g f.w.) of asparagus spears (cv. ‘Grolim’) as affected by spear tip color (white or violet) and spear portion: apical (0–7), middle (7–14) and basal (14–21 cm from the tip).

**Spear portion (cm)**	**Total phenolics**		**Total flavonoids**		**Ascorbic acid**		**DPPH**		**FRAP**	
White spears										
0–7	0.393	b	0.236	b	26.50	b	13.42	b	34.79	b
7–14	0.254	c	0.147	c	15.95	cd	6.23	c	13.85	c
14–21	0.266	c	0.139	c	15.03	de	4.99	c	14.75	c
Violet spears										
0–7	0.502	a	0.430	a	35.17	a	22.38	a	68.33	a
7–14	0.206	d	0.161	c	16.74	c	5.73	c	12.37	c
14–21	0.244	c	0.157	c	14.60	e	5.52	c	13.89	c

Each value is the mean of six replicates. Different letters at each column denote significant differences between means (Duncan’s multiple range test, P < 0.05).

**Table 5.** Total phenolic (mg gallic acid equivalents/g f.w.), total flavonoid (mg rutin equivalents/g f.w.), ascorbic acid (mg/100 g f.w.) content, DPPH (mg ascorbic acid equivalents antioxidant capacity/100 g f.w.) and FRAP (μM/g f.w.) of white asparagus spears (cv. ‘Grolim’) as affected by storage (for 0 or 6 days at 2 °C) and domestic preparation practices (peeling and cooking).

**Preparation practices**	**Total phenolics**		**Total flavonoids**		**Ascorbic acid**		**DPPH**		**FRAP**	
Fresh spears										
Unpeeled	0.251	cd	0.179	c	19.33	a	6.14	b	14.28	b
Peeled	0.252	cd	0.202	bc	17.39	b	5.91	b	16.32	b
Cooked	0.329	a	0.230	b	16.76	bc	17.52	a	58.62	a
Stored spears										
Unpeeled	0.288	bc	0.292	a	15.86	c	5.07	b	18.82	b
Peeled	0.247	d	0.194	bc	15.69	c	5.93	b	17.78	b
Cooked	0.324	ab	0.198	bc	16.43	bc	17.33	a	56.60	a

Each value is the mean of six replicates. Different letters at each column denote significant differences between means (Duncan’s multiple range test, P < 0.05).
